# Assessing emerging technologies from an arms control perspective

**DOI:** 10.3389/frma.2022.1012355

**Published:** 2022-09-12

**Authors:** Maximilian Brackmann, Michèle Gemünden, Cédric Invernizzi, Stefan Mogl

**Affiliations:** ^1^NBC Arms Control Staff Unit, Spiez Laboratory, Federal Office for Civil Protection, Spiez, Switzerland; ^2^Center for Security Studies, ETH Zurich, Zurich, Switzerland; ^3^Chemistry Division, Spiez Laboratory, Federal Office for Civil Protection, Spiez, Switzerland

**Keywords:** dual-use, research of concern, chemical weapons, biological weapons, science policy, security policy

## Abstract

Developments in science and technology improve health and wellbeing of humankind, for example with better methods to detect and treat diseases. However, some advances have led to the development of weapons of mass destruction: chemical and biological weapons. Although banned by international treaties, chemical weapons have been used in recent years in assassinations and the Syrian civil war. Additionally, biological weapons became the subject of recent suspicions and allegations. While not limited to these fields, the so-called dual-use potential—the possibility to apply aspects both with benevolent or malevolent intentions—is especially pronounced in the life sciences. Here, we showcase some areas explored at the conference series Spiez CONVERGENCE that facilitates an exchange between science, arms control and international security. Together, these communities discuss the potential impact of life scientific advances on the Chemical and Biological Weapons Conventions. Enabled by digital technologies, DNA sequencing and synthesis provide the toolbox to (re)construct viruses and cells, which demonstrated invaluable during the COVID-19 pandemic but bear the misuse risk to allow intentionally triggering an outbreak. Open databases and algorithms could be used to generate new chemical weapons. We argue that preventing unintended consequences of life science research while promoting its benefits with responsible science, requires awareness and reflection about unexpected risks of everyone involved in the research process. The strength of the ban of chemical and biological weapons also depends on scientists interacting with policy makers in evaluating risks and implementing measures to reduce them.

## Introduction

The doctor who first synthesized nitroglycerine investigated its vasodilatory properties and its ability to lower blood pressure (Ignarro, 2002). However, nitroglycerine is an explosive that also sparked the development of dynamite by Alfred Nobel, who later initiated the Nobel prize “for the greatest benefit of humankind” realizing that his invention brought much more harm than it did good.^1^,^2^ Fritz Haber was recognized “for the synthesis of ammonia from its elements” which is still instrumental for fertilizer production.^3^ He was later a key driver behind the weaponization of chlorine and phosgene during the First World War (Science History Institute, 2015).

Most scientific research has some aspect that can be used to the benefit of society or misused for nefarious purposes. Whilst by far not being limited to life sciences, dual use is very pronounced in these fields. Developments in science and technology enable the detection, treatment, and cure of many diseases and promote the wellbeing of humankind. At the same time, some of these advances have also led to the development of humanity's worst threats—weapons of mass destruction. Fortunately, international treaties now completely ban chemical and biological weapons.^4^,^5^

Even though they are banned, chemical weapons have regained attention as they have been used over the last years in attempted but also successful assassinations and in the civil war in Syria (Steindl et al., 2021).^6^,^7^ Recently, suspicions and allegations on the development of biological weapons have also been raised more fiercely.^8^,^9^

Fast developments in science and technology and an increasingly volatile international security environment not only emphasize the need for continuous monitoring of scientific advances, but also for raising awareness amongst scientists in the dual-use aspects of their research. This holds true for the knowledge gained, but also the methodology used. Both could potentially be used to construct new chemical or biological weapons (CBW), to modify known agents, or to circumvent existing arms control regimes.

Our intention here is to highlight some topics from a conference series looking at scientific advances from an arms control and dual-use perspective.

## Spiez CONVERGENCE facilitates a dialogue between science, industry and policy making

In 2014, Switzerland started the conference series “Spiez CONVERGENCE” which aims to monitor and assess if and how advances in chemistry and biology impact the Chemical Weapons Convention (CWC, Table 1) and the Biological and Toxin Weapons Convention (BTWC, Table 1) with a particular focus on the interface between biology and chemistry, i.e., their convergence.^10^ This year, the conference series marks its fifth edition and approaches its tenth birthday in 2024. We would like to use this opportunity to review its characteristics, its course and highlight some prime examples that have been discussed.

**Table 1:** The CWC and the BTWC ban entire categories of weapons of mass destruction.

	**The Biological and Toxins Weapons Convention (BTWC or BWC)**	**The Chemical Weapons Convention (CWC)**
	**The Convention on the Prohibition of the Development, Production and Stockpiling of Bacteriological (Biological) and Toxin Weapons and on Their Destruction**	**The Convention on the Prohibition of the Development, Production, Stockpiling and use of Chemical Weapons and on their Destruction**
Opening for signature	1972	1993
Entry into force	1975	1997
Membership	184 States Parties Four Signatory States (not ratified) nine Non-Signatory States	193 States Parties one Signatory State (not ratified) three Non-Signatory States
General Objective	The BTWC has the objective “to exclude completely the possibility of bacteriological (biological) agents and toxins being used as weapons” (see text footnote 6). States Parties are obliged to destroy any stockpiles of biological weapons or delivery systems, prevent proliferation and provide assistance in case of a breach of the Convention (see Article I). Unlike the CWC, the BTWC lacks a verification regime.	The CWC has the objective “to exclude completely the possibility of the use of chemical weapons” (see Article I) (see text footnote 7). It achieves this, inter alia, through its verification regime, which is implemented by the Organization for the Prohibition of Chemical Weapons (OPCW). Under the auspices of the OPCW, more than 99 % of declared stockpiles of chemical weapons have been verifiably destroyed.
First Articles	“*Article I: Each State Party to this Convention undertakes never in any circumstances to develop, produce, stockpile or otherwise acquire or retain:* *(1) microbial or other biological agents, or toxins whatever their origin or method of production, of types and in quantities that have no justification for prophylactic, protective or other peaceful purposes;* *(2) weapons, equipment or means of delivery designed to use such agents or toxins for hostile purposes or in armed conflict.”* (see text footnote 6)	*Extract of Article I:* “*General Obligations* *1. Each State Party to this Convention undertakes never under any circumstances:* *(a) To develop, produce, otherwise acquire, stockpile or retain chemical weapons, or transfer, directly or indirectly, chemical weapons to anyone;* *(b) To use chemical weapons;* *(c) To engage in any military preparations to use chemical weapons;* *(d) To assist, encourage or induce, in any way, anyone to engage in any activity prohibited to a State Party under this Convention.”* (see text footnote 7)
Definition of the types of weapons	The BTWC does not further specify the definition of biological weapons beyond Article I (see above). Biological agents such as bacteria, viruses, fungi, or toxins are considered as biological weapons if they were produced or released with the intention to cause harm in humans, animals, or plants. This could also be the intentional use of insect plagues or overdoses of hormones.	Chemical Weapons are (Article II): “*(a) Any toxic chemical that can cause harm to humans or animals through its chemical action on life processes, and its precursors* *(b) Munitions and devices specifically designed to cause death or other harm through the toxic properties of those toxic chemicals* *(c) Any equipment specifically designed for use directly in connection with the employment of munitions and devices.”* (see text footnote 7) The CWC's Annex on Chemicals lists toxic chemicals in three Schedules for the implementation of the verification measures of the Convention.

Spiez CONVERGENCE is jointly organized by Spiez Laboratory, the Swiss Federal Institute for NBC-Protection, and the Center for Security Studies at ETH Zurich, with support from the Federal Department of Foreign Affairs and the Federal Department of Defence, Civil Protection and Sport^10^. As such, the conference is located at the intersection of science, arms control, and international security. A key value of Spiez CONVERGENCE is to assess new developments in Science and Technology from an arms control perspective and to include scientists, industry representatives and policy experts into such discussion at one table. Researchers from international academic institutions as well as representatives from small start-ups to multi-national corporations contribute to the conference series as invited speakers. Participants range from academic and industry backgrounds to government officials working on chemical and biological weapons non-proliferation and arms control issues. The organizers select topics and invite speakers based on the research's potential to affect arms control treaties.

Bringing together individuals with very diverse backgrounds triggers in-depth discussions about the trajectory of life science research, how these advances potentially challenge existing arms control frameworks, what adjustments might be necessary and about the responsibilities of all actors involved.

The appreciation that the review of disarmament treaties shall include advances in science and technology is not new. The first Review Conference of the BTWC (i.e., its States Parties) was tasked by Art. XII of the BTWC (in 1972) to “…take into account any new scientific and technological developments relevant to the convention...” and the Conference of the States Parties of the CWC (CWC in 1993) stipulated in Art. VIII to “Review scientific and technological developments that could affect the operation of this Convention and, in this context, direct the Director-General to establish a Scientific Advisory Board (…)”.

In contrast to the CWC, the BTWC currently lacks an effective science and technology review process. Even though many proposals to integrate such a process are on the table, they lack consensual support so far. Without an institutionalized review of scientific advances in the life sciences, this task relies entirely on the initiative of States Parties. Within the next year, both treaties will have their Review Conferences to make necessary adjustments.

## Interdisciplinary science challenges arms control

The first Spiez CONVERGENCE conference in 2014 set the scene with the topic clusters “Chemistry Making Biology – Biology Making Chemistry” and “Enabling Technologies”. On one hand, the growing field of Synthetic Biology and Biotechnology depends on the chemical synthesis of biological molecules as building blocks. On the other hand, enzymatic processes are already applied in industrial chemical production processes, blurring the lines between the disciplines. Today, the observed convergence includes many more scientific and engineering disciplines and progress in life sciences is very much interlinked with advancements in instrumentation, engineering and data processing, indicating that these technologies should not be examined in isolation but put into context. Over the years, it was apparent that while many developments require continuous monitoring, others can be set aside, as their direct impact on arms control treaties seems negligible. Importantly, advances in science and technology not only positively or negatively affect societies but also the two before mentioned disarmament treaties. Positive impacts can be the development of novel or more sensitive analytical technologies or novel types of vaccines that not only allow a swift response to pandemics but also to a potential biological weapons attack.

## DNA-sequencing and synthesis enable synthetic biology and novel vaccines

A recurrent topic at Spiez CONVERGENCE has been DNA synthesis and associated technologies like digital genome engineering and synthetic genomics (see text footnote 10). DNA synthesis is a tool providing genetic material, which is as essential for biological research as it is for the development of medical applications, e.g., vaccines. Looking at it in isolation, it allows the production of any (currently rather short) DNA sequence usually by chemical synthesis. However, the integration into a biological system is necessary to incur a biological function, allowing for example the production of a protein, encoded in the DNA sequence. A major bottleneck has always been the limited length of correctly synthesized DNA strands. Advances in DNA synthesis allow increasingly long DNA sequences with higher accuracy and provide the basis to reconstruct whole genomes and to design and synthesize artificial genomes and artificial cells. DNA sequencing and the availability of genomic data are the informational foundation for such approaches. As in DNA synthesis, the abilities to generate longer reads of sequences increase at a very fast pace. Digital technologies like computational algorithms to align, combine and assemble sequences and to optimize them for synthesis are an essential part of this development. They allow the design of artificial genomes and thus facilitate the design and synthesis of artificial cells with defined functions (Venetz et al., 2019).

How this interplay of technologies and data sharing offers great benefits for public health became apparent during the COVID-19 pandemic. Pharmaceutical companies were able to start working on vaccines against the newly identified SARS-CoV-2 virus based on its genetic sequence shortly after the publication of the data (Jackson et al., 2020; Lamb, 2021). Additionally, 33 days after these data sets were accessible in a public database, researchers in Switzerland were able to harvest and work with a reconstructed virus created in their laboratory (Thi Nhu Thao et al., 2020). While public health benefits might be huge, the ability to reconstruct viruses “remotely” also comes with risks and questions on how to tackle potential misuse without hindering any beneficial developments. What if the intention of any actor would be not to prevent an outbreak or investigate countermeasures but to cause an outbreak? This question, however, only becomes relevant once *in vitro* generation of a pathogen is easier than obtaining a natural isolate or if genomic alterations are within the aims of a potential malicious user.

## Computational approaches can predict new potential chemical weapons

Advances in robotics and ever faster and more accurate instrumentation lead to increasing amounts of data, which are usually deposited in open databases. Open and free availability of data accelerates and facilitates research in many fields and opens up new research opportunities that have been hampered before by little or low quality data. The fields that benefit the most from the growing amount of high-quality data are machine learning and artificial intelligence. In previous instances of the conference, omics and multi-omics approaches were discussed; however, major hurdles for the effective integration of different datasets have hindered the efficient interpretation of the vast amount of knowledge contained in these databases.

With their invitation to the conference, speakers often feel encouraged to reflect about potential dual-use or direct misuse risks of their technology and research ahead of the conference. In 2021, a speaker presented how a team in his company adapted previously developed algorithms to predict new pharmaceuticals in order to generate new potential nerve agents (Urbina et al., 2022a). The researchers demonstrated that by using publicly available data, their algorithms depicted thousands of chemical structures with similarity to VX and predicted to be even more toxic. VX too, was among the predicted compounds.

While these developments serve as a wake-up call, they also should be considered in a wider context (Urbina et al., 2022b; Blum, 2022). These chemicals, if used as weapons, are still considered to be chemical weapons. Furthermore, synthetic routes for “traditional” chemical warfare agents are known, partially published and thus easier to use than to establish novel strategies (Blum, 2022). However, the international research and disarmament community must monitor and stay aware of these developments.

## Researchers contribute to the assessment and gain new perspectives on their work

Contributing to the conference and being confronted with the question “What harm could your research do?” triggered many participants to re-think their research and to approach it from a different angle. The dual-use aspect is often not directly obvious, especially to the researchers themselves, as they approach their projects with beneficial results in mind.

As eye-opening and scary some developments seem to be at first sight, the multiple perspectives from industry, academia and policy-making also aid in a realistic assessment of their potentials. Similarly, revisiting and rediscussing technologies proved to be valuable, allowing developments and the associated initial concerns to be set into a broader context. It also provides for a more realistic assessment of potential risks and how they evolve over time when the capabilities of the technologies become more apparent. As an example, advances in additive manufacturing or 3D-printing initially led to concerns that the technology could provide access to equipment that would enable the production of biological and/or chemical weapons, circumventing export control measures that aim at restricting access to certain materials. So far, the capabilities of this technology have not evolved in a way that was initially feared.

## Discussion

More and more available data, acquired with standardized and quality-assured protocols, and better algorithms to analyze them will most certainly equip us with many new and promising insights. Large-scale, multi-center projects, e.g., the Human Proteome or the Beyond 1 Million Genomes projects will also yield insights whose impact we cannot yet completely anticipate in both, their benefits but also their misuse potential (Adhikari et al., 2020).^11^ However, the earlier the risks are considered, the easier they can be mitigated.

Digitalization and artificial intelligence have matured to a state where they immensely advance and influence a wide range of scientific disciplines. Combining data from different sources facilitates the transformation of ideas from the digital space into the physical world, to designing artificial minimal cells or to predicting novel molecules with envisioned properties.

Without doubt, the overwhelming majority of research and development is conducted in good faith and to benefit humanity. Nevertheless, the potential negative aspects and impacts must be considered by everybody involved in the research process—from funders and scientists to publishers—to allow and support research of benefit for the public, while considering potential wider implications.

While research ethics and research integrity has been included in many curricula, teaching responsible science is often neglected. Some universities developed and now require the signing of a Code of Conduct for responsible behavior. For those institutions who are willing to move in the same direction, the WHO developed a Guidance for “Responsible life sciences research for global health security”. Furthermore, Tianjin University and the Johns Hopkins University, together with the InterAcademy Partnership developed the “Tianjin Biosecurity Guidelines for Codes of Conduct for Scientists”. For chemists, “The Hague Ethical Guidelines” offer directions for the responsible conduct of chemistry. The WHO and Tianjin guidelines were developed to be adaptable to the various requirements of institutions and their countries. While they clearly represent a step in the right direction, we also saw that the intention behind a project is often less of a problem than the awareness about the dual-use potential itself.

## Data availability statement

The original contributions presented in the study are included in the article/supplementary material, further inquiries can be directed to the corresponding author/s.

## Author contributions

MB and MG conceptualized and drafted the manuscript. CI and SM substantially contributed to refining the article. All authors contributed to the article and approved the submitted version.

## Conflict of interest

The authors declare that the research was conducted in the absence of any commercial or financial relationships that could be construed as a potential conflict of interest.

## Publisher's note

All claims expressed in this article are solely those of the authors and do not necessarily represent those of their affiliated organizations, or those of the publisher, the editors and the reviewers. Any product that may be evaluated in this article, or claim that may be made by its manufacturer, is not guaranteed or endorsed by the publisher.

## Footnotes

^1^*Full Text of Alfred Nobel's Will*. NobelPrize.org. Available from: https://www.nobelprize.org/alfred-nobel/full-text-of-alfred-nobels-will-2/ (accessed May 18, 2022).

^2^*Alfred Nobel's Life*. NobelPrize.org. Available from: https://www.nobelprize.org/alfred-nobel/biographical-information/ (accessed May 18, 2022).

^3^*The Nobel Prize in Chemistry 1918*. NobelPrize.org. Available from: https://www.nobelprize.org/prizes/chemistry/1918/summary/ (accessed May, 18, 2022).

^4^*UNODA Treaties*. Available from: https://treaties.unoda.org/t/bwc (accessed May 18, 2022).

^5^OPCW. *Chemical Weapons Convention*. Available from: https://www.opcw.org/chemical-weapons-convention (accessed May 18, 2022).

^6^OPCW (2016). *Note by the Technical Secretariat: Report of the OPCW Fact-Finding Mission in Syria Regarding the Incident of the Alleged Use of Chemicals as a Weapon in Kafr Zeita, Syrian Arab Republic*, 53.

^7^OPCW. *OPCW Issues Report on Technical Assistance Requested by the United Kingdom. OPCW*. Available from: https://www.opcw.org/media-centre/news/2018/04/opcw-issues-report-technical-assistance-requested-united-kingdom (accessed May 18, 2022).

^8^*United Nations Not Aware of Any Biological Weapons Programmes, Disarmament Chief Affirms as Security Council Meets to Address Related Concerns in Ukraine*. Meetings Coverage and Press Releases. Available from: https://www.un.org/press/en/2022/sc14827.doc.htm (accessed May 18, 2022).

^9^*United Nations Unaware of Any Biological Weapons Programmes in Ukraine, Top Disarmament Official Affirms, as Security Council Considers New Claims by Russian Federation*. Meetings Coverage and Press Releases. Available from: https://www.un.org/press/en/2022/sc14890.doc.htm (accessed May 18, 2022).

^10^Spiez CONVERGENCE. *Labor Spiez*. Available from: https://www.spiezlab.admin.ch/de/home/meta/refconvergence.html (accessed May 18, 2022).

^11^Beyond One Million Genomes (B1MG) project. Available from: https://b1mg-project.eu/ (accessed May 18, 2022).
